# Middle Georgia Medical Society

**Published:** 1872-02

**Authors:** B. F. Rudicil, R. F. Wright

**Affiliations:** Chairman.; Barnesville, Georgia; Secretary.; Barnesville, Georgia


### MIDDLE GEORGIA MEDICAL SOCIETY.

BARNESVILLE, GEORGIA, January 31, 1872.

At a called meeting of the members of the medical profession residing in the counties of Pike, Monroe and Upson, Dr. B. F. Rudicil was requested to act as Chairman, and Dr. R. F. Wright as Secretary.

Dr. McDowell stated that the object of the convocation was to organize a society for the mutual benefit of the medical fraternity, and for the prosperity and progress of medical science.

On motion of Dr. Strother—

*Resolved*, That this Society be known as the Middle Georgia Medical Society, and that it be regarded simply as an auxiliary to the Georgia State Medical Association.

On motion, the names of members were enrolled:

C. S. Leseur, M.D.; J. O. Hunt, M.D.; T. R. Kendall, M.D.; H. Perdue, M.D.; B. F. Rudicil, M.D.; A. G. Harp, M.D.; G. M. McDowell, M.D.; J. O. Holloway, M.D.; C. S. Strother, M.D.; S. H. Gray, M.D.; S. H. Shi, M.D.; J. E. Cook, M.D.; J. Farmer, M.D.; R. F. Wright, M.D.

On motion—

*Resolved*, That the regular meetings of this Society shall be held bi-monthly, and that the first shall be in Forsyth, on the first Wednesday in March, 1872.

*Resolved*, That the meetings shall be held alternately at the most convenient points within our sphere of action.

On motion, the Chairman appointed a committee—consisting of Drs. McDowell, Kendall and Perdue—to frame a constitution and by-laws, the same to be submitted at the first regular meeting. Also, a committee of five—consisting of Drs. Leseur, Hunt, Holloway, Kendall and Wright—to prepare a schedule of fees, and present the same for consideration at the meeting in March.

On motion, Dr. Leseur was requested to deliver an address on the "Mutual Obligations of Physicians," at the first regular meeting of the Society; and Dr. Rudicil solicited to act as Chairman until its permanent organization—meanwhile, doing all in his power to promote the interests of the body.

On motion—

*Resolved*, That these proceedings be published in the ATLANTA MEDICAL AND SURGICAL JOURNAL.

B. F. RUDICIL, Chairman.

R. F. WRIGHT, Secretary.

